# Clinical and Metabolic Characteristics among Mexican Children with Different Types of Diabetes Mellitus

**DOI:** 10.1371/journal.pone.0168377

**Published:** 2016-12-16

**Authors:** María Lola Evia-Viscarra, Rodolfo Guardado-Mendoza, Edel Rafael Rodea-Montero

**Affiliations:** 1 Department of Pediatric Endocrinology, Hospital Regional de Alta Especialidad del Bajío, León, Guanajuato, México; 2 Department of Research, Hospital Regional de Alta Especialidad del Bajío, León, Guanajuato, México; Baylor College of Medicine, UNITED STATES

## Abstract

**Background:**

Current classification of diabetes mellitus (DM) is based on etiology and includes type 1 (T1DM), type 2 (T2DM), gestational, and other. Clinical and pathophysiological characteristics of T1DM and T2DM in the same patient have been designated as type 1.5 DM (T1.5DM).

**Objectives:**

The aim of this study was to classify pediatric patients with DM based on pancreatic autoimmunity and the presence or absence of overweight/obesity, and to compare the clinical, anthropometric, and biochemical characteristics between children in the different classes of DM.

**Methods:**

A sample of 185 patients, recruited (March 2008-April 2015) as part of the Cohort of Mexican Children with DM (CMC-DM); ClinicalTrials.gov, identifier: NCT02722655. The DM classification was made considering pancreatic autoimmunity (via antibodies GAD-65, IAA, and AICA) and the presence or absence of overweight/obesity. Clinical, anthropometric and biochemical variables, grouped by type of DM were compared (Kruskal-Wallis or chi-squared test).

**Results:**

The final analysis included 140 children; 18.57% T1ADM, 46.43% T1BDM, 12.14% T1.5DM, and 22.86% T2DM. Fasting C-Peptide (FCP), and hs-CRP levels were higher in T1.5DM and T2DM, and the greatest levels were observed in T1.5DM (p<0.001 and 0.024 respectively).

**Conclusions:**

We clearly identified that the etiologic mechanisms of T1DM and T2DM are not mutually exclusive, and we detailed why FCP levels are not critical for the classification system of DM in children. The findings of this study suggest that T1.5DM should be considered during the classification of pediatric DM and might facilitate more tailored approaches to treatment, clinical care and follow-up.

## Introduction

Diabetes mellitus (DM) is characterized by hyperglycemia resulting from defects in insulin secretion and/or insulin resistance in target tissues.[1] The current classification of DM is primarily based on etiology and includes type 1 DM (T1DM), type 2 DM (T2DM), gestational diabetes, and other types of DM. Traditionally, T1DM is a condition that affects lean children or adolescents and young adults. The pathophysiology of T1DM ultimately results in absolute insulin deficiency and hallmark symptoms such as polyuria and polydipsia, with diabetic ketoacidosis (DKA) presenting in approximately 30% of patients.[2] Patients with T1DM require exogenous insulin replacement. Type 1 autoimmune DM (T1ADM) is characterized by β-cell self-destruction. Approximately 70–90% of newly diagnosed cases of T1DM correspond to T1ADM.[3] These patients are identified by the presence of at least one of the following antibodies: anti-islet cell antibodies (AICA), anti-insulin antibodies (IAA), antibodies against glutamic acid decarboxylase 65 (GAD-65), insulinoma-associated autoantigen 2 (IA-2 or ICA512) and antibodies against Zinc transporter 8 (ZnT8).[4] In contrast, patients with type 1 idiopathic DM (T1BDM) do not exhibit signs of self-autoimmunity. T2DM is characterized by obesity, insidious onset, family history of T2DM, residual insulin secretion,[5] and the absence of antibodies against β-cells. In this context, hyperglycemia results from defects in both insulin secretion and insulin function, and most patients with T2DM are treated with oral medications.[6] However, patients presenting with the clinical and pathophysiological characteristics of both T1DM and T2DM have previously been reported. This presentation has been designated type 1.5 DM (T1.5DM), although it is not included in the American Diabetes Association (ADA) classification system.[7,8] T1.5DM has also been referred to as double diabetes or hybrid diabetes.[9]

The prevalence of T2DM in children and adolescents has increased substantially over the past three decades primarily due to the increasing prevalence of pediatric obesity.[10] Children with T1DM (characterized by autoimmune or idiopathic etiology) that have obese family members and/or a family history of T2DM might exhibit clinical features of both types of DM, especially if these children are obese.[11] Clinicians are well aware that the increase in pediatric obesity and T2DM, especially in the Hispanic population, confounds the appropriate clinical diagnosis of children with diabetes.[7,12] This issue has become increasingly problematic, and it is unclear if these patients will have different long-term prognoses, will respond differently to oral medication and insulin regimens, or if they will respond better to a more aggressive or conservative therapeutic approach. Therefore, there is a need to characterize and clearly define the distinct features of nonconventional types of pediatric diabetes.

The aim of this study was to classify pediatric patients with DM based on pancreatic autoimmunity and the presence or absence of overweight/obesity, and to compare the clinical, anthropometric, and biochemical characteristics between children in the different classes of DM.

## Material and Methods

### Patients

A cross-sectional sample of 185 children and adolescents (boys and girls) from 1 to 17 years of age were recruited in the Pediatric Diabetes Clinic (PDC) of the third-level Mexican High-Specialty Regional Bajío Hospital (Hospital Regional de Alta Especialidad del Bajío, HRAEB) located in León, Guanajuato (México) between March 2008 and April 2015. All participants were Mexican-Hispanic. Patients were included in the study if they had recently been diagnosed with DM and had verified pancreatic autoimmunity. Exclusion criteria were neonatal or secondary diabetes (i.e., diabetes associated with the use of steroids or stressful situations).

### Ethics statement

This study is a part of the protocol included in the “Cohort of Mexican Children with Diabetes (CMC-DM)”. CMC-DM is registered with ClinicalTrials.gov, identifier: NCT02722655. The study protocol was reviewed and approved by the Research Committee of the High-Specialty Regional Bajio Hospital and by the Ethics Committee of the High-Specialty Regional Bajio Hospital. Approval number: CI-HRAEB-2015-018. The patients and their parents signed a written informed agreement and consent form, respectively, when they were enrolled in the study.

### Clinical assessments

A physical examination was conducted by a pediatric endocrinologist and included an acanthosis nigricans evaluation based on a modified quantitative scale,[13] described by Burke et al., and puberty staging according to the Tanner Scale. Systolic and diastolic blood pressure (SBP and DBP) were manually measured twice in the right arm using a sphygmomanometer (Riester, Jungingen, Germany) with the appropriate sized cuff while the subject was resting, and the average of the two measurements was used in the analysis. Hypertension was defined as SBP or DBP in the 95^th^ percentile or greater for sex, age and height.[14]

### Anthropometric assessments

Body weight was measured while the children wore light clothing and no shoes. Height was measured with the children standing in an upright position using a Seca stadimeter. Body mass index (BMI) was calculated dividing body weight (kg) by the square of height (m). Body weight, height and BMI percentiles were determined for age and sex according to the Centers for Disease Control (CDC) growth charts. Abdominal waist circumference (WC) was measured in the middle axillary line at the point between the lower edge of the ribs and the top of the iliac crest, and the corresponding percentiles were calculated according to data tables pertaining to Mexican children.[15] Overweight/obese children were defined as children <2 years old with a BMI in the 95^th^ percentile or greater and children aged 2 years or older with a BMI in the 85^th^ percentile or greater.

### Biochemical assessments

Blood samples were collected after an overnight fast when glucose metabolism was stable and the children did not present with acute illness or infection. Fasting C-peptide (FCP) was measured using a chemiluminescence microparticle immunoassay (ARCHITECT SYSTEM, Abbott, IL, USA). Intra- and inter-assay coefficients of variation were less than 10%. The pancreatic β-cell reserve was defined as FCP ≥0.2 nmol/L.[16] Serum total cholesterol (TC), high-density lipoprotein cholesterol (HDL-C) and triglycerides (TG) were measured using dry chemistry with colorimetric methods (Vitros 3350; Ortho Clinical Diagnostic, Johnson & Johnson). Low-density lipoprotein cholesterol (LDL-C) levels were calculated using the Friedewald formula.[17] HbA1C was determined using high-performance liquid chromatography with a DS-5 Analyzer (Drew Scientific, Inc. Miami, FL, USA). High-sensitivity C-reactive protein (hs-CRP) was measured by quantitative immunoturbidimetric determination (CRP Vario 6K26-30 and 6K26-41; Abbott Laboratories Inc. USA).

Insulin sensitivity (IS) was calculated using the equation developed and validated with the euglycemic-hyperinsulinemic clamp used in the SEARCH diabetes in youth study.[18] The following equation was used to measure IS: IS = exp [4.64725–0.02032 x waist (cm) - 0.09779 x [HbA1C(%)] - 0.00235 x TG (mg/dL)]. According to this surrogate measurement, insulin resistance (IR) was defined as IS <8.15 and no insulin resistance (NIR) was defined as IS ≥8.15.[19] FCP levels were adjusted by IS; FCP when IS <8.15 and FCP when IS≥8.15.

### Pancreatic autoimmunity

Antibodies against glutamic acid decarboxylase 65 (GAD-65) were measured using an ELISA kit (from AccuDiagTM, CA, USA) with a sensitivity and specificity of 85% and 87.1%, respectively (antibody-positive was defined as levels >1.05). Anti-insulin antibodies (IAA) were measured using ELISA kits (BioSystems S.A. Barcelona, Spain) with a sensitivity and specificity of 86.7% and 98.7%, respectively (antibody-positive was defined as levels >10 U/mL). Anti-islet cell antibodies (AICA) were evaluated using indirect immunofluorescence methods (monkey pancreas section, BioSystems S.A. Barcelona, Spain) with a sensitivity and specificity of 65% and 100%, respectively. Pancreatic autoimmunity was defined as at least one of three autoantibody-positive results.

### Diagnosis and classification of diabetes

DM was diagnosed based on ADA criteria.[1] The classification of DM types was made as follows:

- T1ADM: non-overweight/obese and positive pancreatic autoimmunity,
- T1BDM: non-overweight/obese and negative pancreatic autoimmunity,
- T2DM: overweight/obese and negative pancreatic autoimmunity,
- T1.5DM: overweight/obese and positive pancreatic autoimmunity.

### Statistical analysis

Data were analyzed using R statistical software.[20] Descriptive statistics were calculated for clinical, anthropometric and biochemical variables grouped by the type of DM, and the values were compared using the Kruskal-Wallis test or the chi-squared test depending on the variable type. The sample size of each group allowed for the detection of a ≥10% difference in any assessment (type I error alpha = 0.05 and type II error beta = 0.80). In all cases, 95% confidence intervals were constructed, and an alpha = 0.05 was considered significant.

## Results

Among the 185 patients with pediatric DM recruited for the study, 45 patients were excluded due to the following reasons: neonatal DM (2), secondary DM (8), and lack of a pancreatic autoimmunity evaluation (35); the pancreatic antibodies were requested in this 35 patients but they did not continue with their follow up in our hospital. The final analysis included 140 children with DM comprising 100 (71.43%) females and 40 (28.57%) males. The mean age at the onset of DM was 9.20 ± 3.71 years (range: 1.18–16.95 years), the mean duration of DM was 1.15 ± 1.78 years. The general characteristics of the study population are provided (Table 1). Overall, the presence of overweight/obesity among the patients was 35.00%. In addition, patients tested positive for the following autoantibodies: GAD-65 (5/97 = 5.15%), IAA (3/97 = 3.09%), and AICA (37/140 = 26.43%).

**Table 1:** Characteristics of the study population.

Variable	n = 140	
Sex		
Male, n (%)	40 (28.57)	
Female, n (%)	100 (71.43)	
Age at onset of DM (years)	9.20 (3.71)	
DKA	55 (39.29)	
Tanner		
1 (prepubescent), n (%)	49 (35.00)	
2–5 (pubescent), n (%)	91 (65.00)	
Hypertension		
No, n (%)	131 (93.57)	
Yes, n (%)	9 (7.13)	
Acanthosis (0–16)	2.47 (4.08)	
0: No, n (%)	82 (58.57)	
1–16: Yes, n (%)	58 (41.43)	
BMI category		
Lean, n (%)	91 (65.00)	
Overweight/obese, n (%)	49 (35.00)	
Positive anti-islet autoantibodies		
GAD-65, n (%)	5 (5.15)	n = 97
IAA, n (%)	3 (3.09)	n = 97
AICA, n (%)	37 (26.43)	

Unless otherwise indicated, values are presented as the mean (standard deviation).

**Fig 1:**
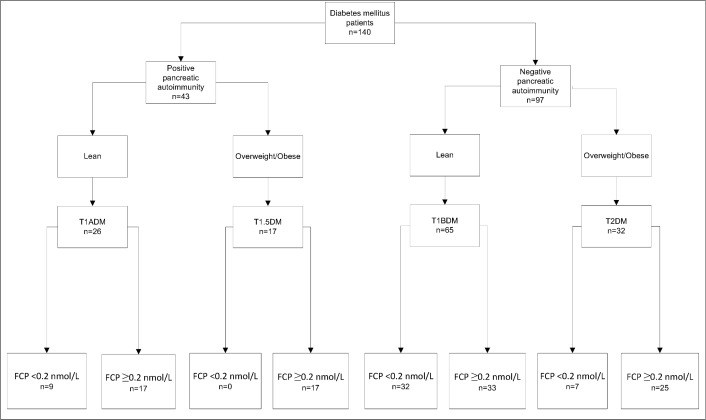
Classification system of diabetes mellitus in pediatric patients.

The DM classification system was based on pancreatic autoimmunity and body composition (lean or overweight/obese). This system classifies patients into one of four groups (Fig 1) as follows: T1ADM (26 patients, 18.57%), T1BDM (65 patients, 46.43%), T1.5DM (17 patients, 12.14%) and T2DM (32 patients, 22.86%).

The FCP levels were analyzed as an indirect measurement of pancreatic β-cell reserve and the proportion of patients with pancreatic β-cell reserves within each study group were as follows: T1.5DM (17/17, 100%), T2DM (23/32, 71.8%), T1ADM (16/26, 61.5%) and T1BDM (31/65, 47.7%).

The clinical and anthropometrical data (Table 2 and Table 3 respectively) illustrate that duration of DM was higher in patients with T1DM (p = 0.031), and that both the age of onset of DM and the Tanner stage were greater in patients with T2DM (p = 0.004 and p = 0.006, respectively). DKA was more frequently observed in patients with T1ADM, but the difference compared with the other groups was not statistically significant (p = 0.061). At the time of sampling, insulin use was more frequently observed in patients with T1DM (p<0.001) but insulin dose was no significant different between groups (p = 0.901), and metfomin use was more frequently observed in patients with T2DM and T1.5DM (p<0.001). A family history of overweight/obesity and T2DM were more frequently observed in the T1.5DM and T2DM groups (p = 0.008 and p = 0.011, respectively), and acanthosis was more frequently observed in the T1.5DM and T2DM groups (p<0.001) compared with the other groups. There were no significant differences in DBP and hypertension between the different groups (p = 0.709 for both groups). However, SBP was significantly higher in patients with T2DM (p = 0.012). With respect to the anthropometric evaluation, WC and WHtR were significantly greater in the T2DM and T1.5DM groups (p<0.001 for both groups).

**Table 2:** Clinical characteristics of the study population grouped by type of DM.

Variable	Overall n = 140		T1ADM (Autoimmune) n = 26		T1BDM (Idiopathic) n = 65		T1.5DM n = 17		T2DM n = 32		p-value^a^
Sex											p = 0.331
Male, n (%)	40 (28.57)		6 (23.08)		17 (26.15)		8 (47.06)		9 (28.13)		
Female, n (%)	100 (71.43)		20 (76.92)		48 (73.85)		9 (52.94)		23 (71.88)		
Duration of DM (years)	1.15 (1.78)		1.07 (1.61)		1.58 (2.12)		0.42 (0.46)		0.71 (1.37)		p = 0.031^b^
Age at onset of DM (years)	9.20 (3.71)		7.87 (3.75)		8.60 (3.77)		9.85 (2.72)		11.17 (3.29)		p = 0.004^b^
DKA, n (%)	55 (39.29)		15 (57.70)		25 (38.50)		8 (47.10)		7 (21.90)		p = 0.061
Insulin use, n(%)	118 (84.30)		25(96.20)		64 (98.50)		11 (64.70)		18 (56.20)		P<0.001^b^
Insulin dose (U/kg/day)	0.88 (0.41)	n = 118	0.91 (0.43)	n = 25	0.88 (0.44)	n = 64	0.89 (0.28)	n = 11	0.81 (0.38)	n = 18	p = 0.901
Metformin use, n(%)	30 (21.40)		2 (7.70)		6 (9.20)		5 (29.4)		17 (53.1)		p<0.001 ^b^
Overweight/obese family history, n (%)	102 (77.27)	n = 132	15 (62.50)	n = 24	44 (70.96)	n = 62	16 (100.00)	n = 16	27 (90.00)	n = 30	p = 0.008^b^
T2DM family history, n (%)	111 (79.29)		17 (65.40)		48 (73.90)		15 (88.20)		31 (96.90)		p = 0.011^b^
Acanthosis (0–16)	2.47 (4.08)		0.65 (1.65)		0.69 (1.48)		5.18 (5.94)		6.13 (4.76)		p<0.001^b^
Acanthosis											p<0.001^b^
0: No, n (%)	82 (58.57)		20 (79.92)		50 (76.92)		6 (35.29)		6 (18.75)		
1–16: Yes, n (%)	58 (41.43)		6 (23.08)		15 (23.08)		11 (64.71)		26 (81.25)		
Tanner (1–5), median	2		2		2		2		3.50		p = 0.006^b^
Tanner											p = 0.147
1 (Prepubescent), n (%)	49 (35.00)		12 (46.15)		25 (38.46)		7 (41.18)		5 (15.63)		
2–5 (Pubescent), n (%)	91 (65.00)		14 (53.85)		40 (61.54)		10 (58.82)		27 (84.38)		
SBP (mmHg)	100.40(13.67)		100.38 (11.40)		97.58 (13.09)		98.47 (17.31)		107.16 (12.59)		p = 0.012^b^
SBP percentile											p = 0.668
<90th (1), n (%)	121 (86.43)		23 (88.46)		58 (89.23)		15 (88.24)		25 (78.13)		
90th to <95th (2), n (%)	10 (7.14)		1 (3.85)		5 (7.69)		1 (5.88)		3 (9.38)		
≥95th (3), n (%)	9 (6.43)		2 (7.69)		2 (3.08)		1 (5.88)		4 (12.50)		
DBP (mmHg)	59.32 (9.70)		57.81 (8.95)		58.66 (9.24)		60.59 (11.30)		61.22 (10.38)		p = 0.282
DBP percentile											p = 0.709
<90th (1), n (%)	127 (90.71)		24 (92.31)		60 (92.31)		15 (88.24)		28 (87.5)		
90th to <95th (2), n (%)	6 (4.29)		1 (3.85)		1 (1.54)		1 (5.88)		3 (9.38)		
≥95th (3), n (%)	7 (5.00)		1 (3.85)		4 (6.15)		1 (5.88)		1 (3.13)		
Hypertension (SBP ≥95th or DBP ≥95th percentile), n (%)	9 (6.43)		2 (7.69)		2 (3.08)		1 (5.88)		4 (12.50)		p = 0.709

Unless otherwise indicated, values are given as the mean (standard deviation).

^a^Kruskal-Wallis test with 3 degrees of freedom or chi-square test with 3 degrees of freedom according to the type of variable.

^b^Significant p-value.

**Table 3:** Anthropometric characteristics of the study population grouped by type of DM.

Variable	Overall n = 140		T1ADM (Autoimmune) n = 26		T1BDM (Idiopathic) n = 65		T1.5DM n = 17		T2DM n = 32		p-value^a^
Weight (kg)	39.97 (18.17)		30.53 (13.21)		33.34 (13.23)		49.92 (21.12)		55.85 (16.35)		p<0.001^b^
Weight percentile	57.81 (32.36)		48.11 (24.45)		38.03 (27.68)		90.81 (8.57)		88.35 (11.6)		p<0.001^b^
Height (cm)	137.07 (20.89)		127.93 (22.69)		134.92 (21.93)		140.82 (16.71)		146.89 (14.74)		p = 0.004^b^
Height percentile	39.59 (28.87)		40.51 (27.89)		33.79 (28.23)		52.97 (27.61)		43.52 (29.76)		p = 0.059
BMI (kg/m^2^)	20.11 (4.89)	n = 137	17.65 (2.63)	n = 25	17.42 (2.75)	n = 63	24.14 (5.50)		25.17 (3.73)		p<0.001^b^
BMI percentile	63.99 (31.40)	n = 137	53.85 (29.33)	n = 25	44.59 (26.02)	n = 63	94.29 (3.94)		94.02 (3.86)		p<0.001^b^
WC (cm)	68.91 (14.18)	n = 125	62.29 (11.52)	n = 25	62.15 (8.47)	n = 53	77.10 (17.59)	n = 16	81.57 (11.28)	n = 31	p<0.001^b^
WC percentile											p<0.001^b^
<90th, n (%)	86 (81.90)	n = 105	10 (66.67)	n = 15	44 (100.00)	n = 44	16 (94.12)		16 (55.17)	n = 29	
≥90th, n (%)	19 (18.10)	n = 105	5 (33.33)	n = 15	0 (0.00)	n = 44	1 (5.88)		13 (44.83)	n = 29	
WHtR	0.50 (0.07)	n = 125	0.49 (0.05)	n = 25	0.47 (0.05)	n = 53	0.55 (0.08)	n = 16	0.56 (0.05)	n = 31	p<0.001^b^

Unless otherwise indicated, values are given as the mean (standard deviation).

^a^Kruskal-Wallis test with 3 degrees of freedom or chi-square test with 3 degrees of freedom according to the type of variable.

^b^Significant p-values.

The biochemical characteristics of the study population according to the type of DM (Table 4) illustrates that although there were no significant differences in lipid profiles between the study groups, TG levels (p = 0.124) were higher in the T1.5DM group, and the highest levels were observed in the T2DM group. Similarly, HDL-C levels tended to be lower in the T1.5DM and T2DM groups compared with the other groups (p = 0.074).

**Table 4:** Biochemical characteristics of the study population grouped by type of DM.

Variable	Overall n = 140		T1ADM (Autoimmune) n = 26		T1BDM (Idiopathic) n = 65		T1.5DM n = 17		T2DM n = 32		p-value^a^
Triglycerides (mmol/L)	1.41 (1.01)	n = 136	1.24 (0.82)	n = 25	1.29 (1.01)	n = 63	1.53 (1.07)		1.72 (1.07)	n = 31	p = 0.124
Cholesterol (mmol/L)	4.05 (0.82)	n = 134	3.98 (0.93)	n = 25	4.10 (0.84)	n = 62	4.06 (0.66)	n = 16	4.02 (0.82)	n = 31	p = 0.909
HDL-C (mmol/L)	1.21 (0.37)	n = 130	1.26 (0.39)	n = 25	1.28 (0.36)	n = 61	1.16 (0.36)	n = 14	1.06 (0.33)	n = 30	p = 0.074
LDL-C (mmol/L)	2.24 (0.68)	n = 130	2.22 (0.73)	n = 25	2.24 (0.74)	n = 61	2.35 (0.60)	n = 14	2.20 (0.57)	n = 30	p = 0.745
HbA1C (%)	9.57 (2.53)	n = 115	9.14 (2.32)	n = 23	10.37 (2.60)	n = 54	8.77 (2.09)	n = 13	8.64 (2.36)	n = 25	p = 0.020^b^
FCP (nmol/L)	0.83 (0.91)		0.91 (0.95)		0.55 (0.73)		1.63 (1.05)		0.93 (0.87)	n = 32	p<0.001^b^
Pancreatic reserve											p = 0.001
FCP <0.2 nmol/L, n (%)	48 (34.30)		9 (34.62)		32 (49.23)		0 (0.00)		7 (21.88)		
FCP ≥0.2 nmol/L, n (%)	92 (65.70)		17 (65.38)		33 (50.77)		17 (100.00)		25 (78.13)		
Insulin sensitivity	8.43 (3.29)	n = 108	9.92 (4.07)	n = 22	8.78 (2.89)	n = 48	7.78 (3.12)	n = 13	6.76 (2.68)	n = 25	p = 0.006^b^
Insulin resistance											P = 0.003^b^
IR (IS <8.15), n (%)	48 (44.40)	n = 108	6 (27.30)	n = 22	16 (33.30)	n = 48	8 (61.50)	n = 13	18 (72.00)	n = 25	
NIR (IS ≥8.15), n (%)	60 (55.60)	n = 108	16 (72.70)	n = 22	32 (66.70)	n = 48	5 (38.50)	n = 13	7 (28.00)	n = 25	
FCP adjusted by IS											
FCP when IS <8.15	2.96 (2.94)	n = 48	1.34 (1.99)	n = 6	1.89 (2.34)	n = 16	5.54 (2.81)	n = 8	3.31 (3.06)	n = 18	p = 0.011^b^
FCP when IS ≥8.15	1.88 (2.27)	n = 60	3.17 (3.08)	n = 16	1.28 (1.66)	n = 32	2.48 (2.33)	n = 5	1.28 (1.35)	n = 7	p = 0.036^b^
hs-CRP (mg/dL)	2.07 (3.33)	n = 115	1.43 (1.58)	n = 23	1.65 (2.79)	n = 54	3.82 (7.00)	n = 13	2.64 (2.41)	n = 25	p = 0.024^b^

Unless otherwise indicated, values are given as the mean (standard deviation).

^a^Kruskal-Wallis test with 3 degrees of freedom or chi-square test with 3 degrees of freedom according to the type of variable.

^b^Significant p-values.

Metabolic control, expressed as HbA1C levels, was poorest in patients with T1ADM and T1BDM (p = 0.020). FCP levels were higher in the T1.5DM and T2DM groups, and the greatest levels were observed in the T1.5DM group compared with the other groups (p = 0.001). IS was significantly lower in the T1.5DM and T2DM groups (p = 0.006). FCP adjusted by IS was higher in T1.5DM and T2DM when IS<8.15 (p = 0.011) and was higher in T1ADM and T1.5DM when IS≥8.15 (p = 0.036). hsCRP levels were significantly greater in the T1.5DM and T2DM groups (p = 0.024), with the greatest levels observed in the T1.5DM group.

## Discussion

In the present study, we classified Mexican children with DM and characterized the distinct clinical and biochemical features associated with the different groups. In recent years, the diagnosis and classification of some children with DM has become increasingly difficult, and a significant factor associated with this challenge is the obesity epidemic. To facilitate the appropriate classification of children with DM, some studies have recommended the use of laboratory tests to examine human leukocyte antigen (HLA) genotypes, anti-pancreatic autoantibodies and C-peptide levels.[19,21,22] However, these tests are not readily available in the majority of first-line clinical care centers, and physicians frequently diagnose DM subtypes based on the clinical presentation.

Over the last three decades, the increase in childhood obesity has mirrored the increasing prevalence of T2DM, especially among ethnic minorities.[6] Mexico has a high prevalence of overweight/obesity, with 71.2% of adults and 34.4% of children classified as overweight/obese.[23] Consistent with national trends in pediatric overweight/obesity, in the present study, we observed a high prevalence of overweight/obesity (35.00%) and a high prevalence of relatives with overweight/obesity (77.27%) and T2DM (79.29%) in pediatric patients with DM. Due to the genetic predisposition of Mexicans, overweight/obesity is a good indicator of IR,[24] and although autoimmunity can occur concomitantly with overweight/obesity, assessment of this parameter requires laboratory diagnostics.

In this study, pancreatic autoimmunity was observed in 30.71% of patients, a relatively low rate compared with that observed in other countries (56.60–90.00%).[19,25,26] A low prevalence of anti-GAD-65 and IAAs was observed, at 5% and 3%, respectively. The prevalence of IACAs was relatively higher at 26.43%. These results might be influenced by several factors, including the time elapsed between the diagnosis of DM and the clinical evaluation, variations in sensitivity and specificity of the assays, a relatively lower prevalence of autoimmunity in this population,[27] and the lack of measurement of antibodies against ZnT8, a parameter that has been recently reported to be an indicator of autoimmunity. [28]

In contrast with studies conducted in other countries,[19,25] we evaluated an ethnically homogenous Mexican population, a population with a strong predisposition to T2DM. Previous studies compared the classification system of pediatric DM determined by the clinical criteria evaluated by a pediatric endocrinologist with a classification system incorporating pancreatic autoimmunity, C-peptide,[29] and HLA.[30] In this study, we incorporated two key quantitative etiological markers, overweight/obesity and pancreatic autoimmunity, into a DM classification system, similar to the system proposed by Purushutama et al. in which the overweight/obese variable is the primary factor.[25] The prevalence of T2DM according to the system proposed in this study was 22.80%, which is similar to previous reports for Hispanic children living in North America.[31] Most of the patients in this study were classified as having T1DM, with a cumulative proportion of patients with T1ADM and T1BDM of 64.7%, similar to that reported in other countries.[19,21] The proportion of patients with T1ADM was relatively low (18.57%), primarily due to the low prevalence of pancreatic autoimmunity previously mentioned. However, if some patients in the T1BDM group presented a false-negative result in the pancreatic autoimmunity evaluation, then it would result in an increase in the prevalence of T1ADM in the cohort without altering the prevalence of T1DM and/or invalidating the comparative analysis between the groups. The prevalence of T1.5DM in the patients cohort was 12.14%, which was lower than the prevalence reported in the SEARCH study in which pancreatic autoimmunity and IR were identified in 19.5% of patients.[19]

The prevalence of T1.5DM reported in different studies can vary widely because there is a lack of standardized and precise classification criteria. Some clinical centers do not measure pancreatic autoimmunity in children with clinical characteristics of T2DM; therefore, classification in these cases is primarily based on the clinical presentation. Other studies have not recognized T1.5DM as an independent subtype of DM and instead report these patients as having T2DM accompanied by pancreatic autoimmunity.[32–34]

We were not able to perform HLA genotyping in the patients evaluated in this study. However, these data are not relevant to DM classification because the high risk associated with the HLA genotype applies to only 50.00% of all patients with T1ADM.[19,35] Moreover, the high-risk HLA genotype does not preclude the concomitant presence of IR and/or obesity because these features are observed in T1.5DM patients. In addition, the role of genetic determinants in T1.5DM and T2DM is complex due to the polygenic etiology of IR.

Redondo et al. proposed a classification of DM in children according to the Ketosis-Prone Diabetes classification system previously used in adults.[36] This system groups patients according to autoimmunity and pancreatic β-cell reserve (FCP ≥0.2 nmol/L). The 2-year follow-up demonstrated that the group with higher FCP levels exhibited lower HbA1C. This classification is distinct from the ADA etiological classification in which T1DM or T2DM patients with a pancreatic β-cell reserve might be classified inappropriately because it was recently shown that some patients with T1DM and T2DM might have a residual pancreatic β-cell reserve, primarily demonstrated by measurements of fasting or stimulated C-peptide.

In this context, C-peptide level is a general indicator of endogenous insulin production. C-peptide is nearly undetectable in patients with T1DM and even in patients with T2DM patients, depending on the level of residual endogenous insulin production,[22] duration of DM, honeymoon period, drug treatment, degree of autoimmunity, and IR.

C-peptide level was not included in our classification system because we found that more than half of the patients with T1DM (autoimmune and idiopathic) (50/91 = 54.95%) had pancreatic β-cell reserves and FCP ≥0.2 nmol/L. The proportion of patients with T1DM with pancreatic β-cell reserves increased to 63.74% (58/91) if we applied the cutoff recommended by the SEARCH study,[19] which defined the pancreatic β-cell reserve as FCP ≥0.13 nmol/L. According to this definition, 61.00% of children with T1DM in the SEARCH study had pancreatic β-cell reserves, similar to our observations. In this study, the pancreatic β-cell reserve was observed in 78.13% (25/32) of children with T2DM. In addition, 84.38% (27/32) of these patients presented with FCP ≥0.13 nmol/L and 84.38% with (27/32) FCP ≥0.2 nmol/L. All children classified as having T1.5DM exhibited FCP levels ≥0.2 nmol/L.

However, other groups have reported that absolute insulin deficiency is observed in most, but not all, patients with T1DM.[35] Oram et al. have reported that patients with T1DM produce C-peptide within the first 5 years after the diagnosis of DM.[37] Based on these findings, the “accelerator hypothesis” postulating that DM is a single disease with a variable rate of β-cell loss might be applicable;[38] slow in T2DM (IR) and rapid in T1ADM (autoimmunity).[39] It is possible that the rate of β-cell loss in patients with T1DM depends on other factors in addition to autoimmunity. A honeymoon period has also been observed, and it now seems clear that pancreatic autoimmunity in patients with T1DM does not guarantee an absolute deficiency in insulin production/secretion. In our study only one patient of T1ADM group was in honeymoon period (exogen insulin dose ≤ 0.5 UI/kg/day with HbA1C ≤ 6%). In adult patients, there is evidence indicating that β-cell destruction might lead to a slow progression to a form of autoimmune diabetes characterized by the presence of the autoimmunity observed in T1DM and a clinical presentation at diagnosis reminiscent of patients with T2DM (referred to as latent autoimmune diabetes in adults, LADA).[10,40] When we analyzed FCP adjusted by IS, we found that among IR patients FCP levels were lower in T1DM, and among non IR patients FCP levels were higher in T1ADM and T1.5DM. It is remarkable that FCP levels were higher in T1.5DM independently of IS.

Based on these observations, we agree that FCP levels are not critical parameters for the classification system of DM in children. The FCP level is an indicator of disease progression in T1DM and T2DM, but it is not an etiological marker like pancreatic autoimmunity and obesity.

The accelerator hypothesis proposes that obesity is associated with a decrease in the age of onset of T1DM,[39] however, we found an older age of onset of T1.5DM in children compared with T1DM but a younger age compared with T2DM, a trend that is distinct from T1DM. Patients with T1.5DM exhibit both of the primary pathophysiological features of DM: IR and autoimmunity. Interestingly, in our study, all patients with T1.5DM exhibited residual pancreatic β-cell reserves, implying that these patients exhibit a greater β-cell response then previously presumed, perhaps due to the insulin requirements secondary to obesity and IR. Certainly, the group of patients with T1.5DM had a lower mean DM duration and 35.30% of the subjects of this group did not use insulin, it makes plausible that some of the subjects of the T1.5DM group were likely in the honeymoon period and this fact could have modified their FCP levels. However, there is evidence (clinical and/or biochemical) of IR in all of the subjects of the T1.5DM group.

In our study, there was a greater proportion of female patients with both T1DM and T2DM,[19,26] which differs slightly compared with previous studies.

The system classification divided patients into four groups, and the characteristics of each group were consistent with previous reports: children classified as having T2DM exhibited a similar clinical presentation: a greater frequency of a family history of T2DM and obesity, a more advanced average age of onset and stage of puberty, a lower frequency of DKA, higher WHtR, a greater frequency of acanthosis nigricans, higher SBP and higher frequency of hypertension. These patients exhibited biochemical characteristics similar to those reported to be associated with T2DM: hypertriglyceridemia, relatively low HDL levels, higher hs-CRP levels (an adipose tissue marker of inflammation), lower HbA1C, and an IR classification of IS. All of these features are consistent with the pathological mechanisms of T2DM.

In our study, we did not observe any clinical or biochemical differences between patients with T1ADM and T1BDM, with the exception of the presence of markers of pancreatic autoimmunity. The patients in both groups presented the clinical features of T1DM: an early average age of onset was observed in these patients, with almost half of them diagnosed during pre-pubertal development. In addition, these patients exhibited a higher frequency of DKA at disease onset, a lower WHtR, acanthosis nigricans in very few children according to Burke´s scale, a lower frequency of a family history of obesity and/or T2DM, lower hs-CRP levels, higher HbA1C, lower FCP levels and IS classified as normal.

Children classified in the T1.5DM group were composed of a similar proportion of males and females. This group exhibited an overlap of the clinical and biochemical characteristics similar to those observed T1DM (autoimmunity) and T2DM (IR), consistent with the pathological origins of this disease. These patients were associated with a greater frequency of a family history of obesity (100.00%) and (88.20%) and T2DM, and the average age onset of DM was (9.85 ± 2.72) between that observed in patients with T1DM (7.87 ± 3.75) and T2DM (11.17 ± 3.29). Acanthosis nigricans documented by Burke´s scale was similar in patients with T1.5DM and T2DM (5.18 ± 5.94 vs 6.13 ± 4.76, respectively). Patients with T1.5DM exhibited a lipid profile that was intermediate between that observed in patients with T1DM and T2DM, although no significant differences were observed. HbA1C was higher compared with T2DM but lower compared with T1DM, and all children in this group exhibited the highest FCP levels (>0.2 nmol/L) among the groups, consistent with the residual pancreatic β-cells. hs-CRP levels were higher compared with T2DM, and most of the children were classified as IR (61.7%); however, this was still a lower frequency compared with T2DM and a higher frequency compared with T1DM.

It is important to recognize T1.5DM a distinct subtype of DM in children that is characterized by the co-existence of the etiologic processes of autoimmunity and the peripheral defects in insulin signaling in the same patient, factors that are primarily due to obesity (IR). It is also worth recognizing that the etiologic mechanisms of DM are not mutually exclusive because they can be present in pediatric patients with T1DM and T2DM. Recognizing this phenomenon might improve individualized disease management and identify prognostic factors indicative of a patient’s response to treatment.

Due to the worldwide obesity epidemic observed in recent years, it is likely that some patients diagnosed with T1DM should be reclassified during the course of the disease, particularly when these patients present with weight gain, which might initiate IR. This might represent a factor of the DM classification system that varies over time, suggesting that some patients might be classified differently over the course of the disease and require specific treatment modification.

This study has certain limitations. First, the study was cross-sectional in nature; therefore, causality cannot be inferred. Second, we did not incorporate the detection of autoantibodies against IA-A2 and ZnT8 in the evaluation of pancreatic autoimmunity. Third, no MODY testing was performed. A major strength of the study is that permits the characterization and comparison of the types of DM in Mexican children. Longitudinal prospective studies to characterize the progression of pancreatic autoimmunity and the impact of IR in different types of DM are necessary. In addition, the influence of these pathogenic factors on the evolution of DM and the risk of chronic complications also requires further study. Such studies could provide new insights into the progression of DM and facilitate the identification of therapies that will be the most effective in these children.

## Conclusions

In conclusion, T1DM is the most prevalent type of diabetes observed in Mexican children. However, the prevalence of T2DM was considerably high for a pediatric population. The prevalence of T2DM in native Mexican children is increasing and is now similar to that observed in Mexican children living outside the country. This observation suggests that these similarities are due to genetic and sociological factors. The definition of T1.5DM includes two of the primary factors in the pathogenesis of DM: pancreatic autoimmunity and IR. The recognition of this distinct type of DM might facilitate more tailored approaches to treatment, clinical care and follow-up, as well as help minimize the development of chronic complications. The findings of this study suggest that T1.5DM should be considered during the classification of pediatric DM.
